# Partner phubbing and quality of romantic relationship in emerging adults: testing the mediation role of perceived partner responsiveness and moderation role of received social support

**DOI:** 10.1186/s40359-025-02942-3

**Published:** 2025-06-05

**Authors:** Xin Wang, Momo Su, Lvzhou Ren, Huihui Xu, Xinyi Lai, Jiankang He, Mingxuan Du, Chengjia Zhao, Guohua Zhang, Xue Yang

**Affiliations:** 1 Center for Health Behaviours Research, JC School of Public Health and Primary Care, Faculty of Medicine, The Chinese University of Hong Kong, 5/F, Shatin, New Territories, Hong Kong SAR, China; 2 Research School of Psychology, College of Health and Medicine, Australian National University, Canberra, 2601 Australia; 3 Department of Psychology, School of Mental Health, Wenzhou Medical University, Wenzhou, 325035 China; 4 The Affiliated Kangning Hospital, Wenzhou Medical University, Wenzhou, 325035 China; 5 Key Laboratory of Alzheimer’s Disease of Zhejiang Province, Institute of Aging, Wenzhou Medical University, Wenzhou, Zhejiang China

**Keywords:** Emerging adults, Partner phubbing, Perceived partner responsiveness, Quality of romantic relationship, Received social support

## Abstract

**Background and aims:**

The association between partner phubbing and the quality of romantic relationships (QR) is inconsistent in previous studies. Furthermore, the mechanisms underlying such an association are still unclear. The current study examined the association between partner phubbing and QR, the potential mediating role of perceived partner responsiveness (PPR), and the moderation effects of received social support (RSS) and gender in this association among emerging adults.

**Methods:**

An online cross-sectional survey was conducted among 772 Chinese young adults with regular romantic partners (mean age = 21.54).

**Results:**

The moderated mediation model showed that PPR mediated the negative association between partner phubbing and QR among females but not for males (indirect effect = -0.17, 95% CI [-0.24, -0.10], PM = 70.8%). RSS significantly moderated the positive association between PPR and QR in both females and males. In males, RSS significantly moderated the negative association between partner phubbing and QR and between partner phubbing and PPR.

**Conclusions:**

This is the first study to test both PPR and RSS as potential mechanisms in the association between partner phubbing and QR, which underlines the potential interpersonal implications of partner phubbing among young couples. The results highlight the different associations and paths from partner phubbing to QR in males and females.

**Clinical trial number:**

Not applicable.

## Background

Living in a time when most of our daily activities are carried out via smartphones, people are finding it hard not to check their phones from time to time. On some occasions, it can cause problems. “Phubbing” is a word derived from “phone” and “snubbing”, which describes a behavior where one chooses to focus on their smartphone and snub the person that is physically co-present [1]. In research conducted by McDaniel, Galovan [2], over 72.4% of Americans think using smartphones interferes with their daily lives. In India, 48.8% of respondents endorsed that they were phubbed by others 3–5 times per day [3]. A growing body of literature has elucidated that phubbing behavior was positively associated with smartphone addiction and psychological issues like anxiety and depression [4].

### The association between partner phubbing and the quality of romantic relationship (QR)

Emerging adulthood—a developmental phase spanning from late adolescence to early adulthood—presents significant challenges to cognitive, emotional, and behavioral health [5]. A key developmental task during this period is the formation and maintenance of romantic relationships [6]. Empirical research has shown that emerging adults engaged in high-quality romantic relationships tend to report lower levels of externalizing behaviors and fewer internalizing symptoms [6]. As digital technology becomes increasingly embedded in daily life, emerging adults often rely on it as a primary means of social connection [7]. In this context, phubbing may be particularly detrimental to romantic relationships during emerging adulthood, a time when individuals are still developing relational competencies and emotional regulation skills [6, 7], potentially making them more susceptible to relationship disruptions than individuals in other life stages. QR can be reflected by how positively or negatively a person evaluates his/her relationship with a romantic partner [8]. Higher levels of partner phubbing may cause feelings of being ignored and less intimate [9, 10], which may lead to poor QR. However, the empirical studies have shown conflicting results. For instance, several studies demonstrated that partner phubbing is negatively associated with QR among romantic partners [10–15]. Some research reported non-significant associations [16, 17]. Nevertheless, Çizmeci [18] found that Turkish unmarried women with higher levels of partner phubbing behavior also tend to report higher levels of relationship satisfaction. The cultural orientation among Turkish non-married women may explain this counterintuitive finding. It is reported that Turkish non-married women are more future-oriented in their relationship plans and work harder to maintain their relationships; So, they tend to report high relationship satisfaction [19]. Therefore, the mixed results indicate there may exist mediating or moderating factors that can influence the relationship between partner phubbing and QR. Therefore, more studies are warranted to better understand their underlying mechanisms.

### The mediation role of perceived partner responsiveness

An important mediator that can explain partner phubbing and QR may be the perceived partner responsiveness (PPR). PPR reflects the extent to which individuals believe their partners understand, validate, and care for them [20]. Evidence has shown that PPR is a strong determinant of good QR [21]. A responsive person who responds supportively and empathically in interactions would make his/her partner feel understood, appreciated, and valued, which is a core process of creating good relationships. However, when people are distracted by smartphones and do not express responsive behaviors as their partners expected, their PPR might be affected. An experiment study showed that even having a smartphone around could negatively impact people’s perceived understanding, respect, and responsiveness to the speaker in the conversation [22]. This finding was also demonstrated in a non-experimental study [23]. Hence, PPR would be reduced by phubbing behaviors, which in turn, contribute to poor QR. Indeed, we identified two recent studies reporting that phubbing was indirectly associated with QR through PPR in romantic partners [12, 16]. However, both studies were based on small sample sizes and in the context of Western culture. No study has tested this mediating hypothesis among the Chinese population.

### Received social support and stress-buffer hypothesis

The mediating effect of PPR between partner phubbing and QR may be moderated by received social support (RSS). RSS refers to the quantity of actual support received from one’s important others, including romantic partners. It is conceptually distinct from PPR; while RSS focuses on the actual amount of support received from one’s partner, PPR emphasizes the general perception of one’s partner as responsive, understanding, and empathetic [24]. RSS from one’s romantic partner was also associated with positive emotions and high-quality and stable romantic relationships [25]. Based on the stress buffer hypothesis [26], social support can mitigate the harmful impact of stressors on well-being. In the case of partner phubbing, RSS may buffer the impact of strain due to being ignored, interpersonally excluded, and perceived partner’s unresponsiveness and lack of empathy on QR. Similarly, when RSS is high, individuals may feel more emotionally secure, even if their PPR is low. The high RSS buffers the negative impact of low PPR on QR. When RSS is low, individuals may depend more heavily on PPR for relational satisfaction. In such cases, low PPR would more strongly predict lower QR, because there are no compensatory resources. We did not identify any studies on the buffering effect of RSS on the associations between partner phubbing, PPR, and QR.

Furthermore, the application of the stress-buffering model to gender-stratified effects warrants deeper exploration, drawing on established literature on different gendered patterns in appraisal of stress and seeking and utilization of social resources [27, 28]. Previous research has reported gender differences in attitudes toward phubbing [13], the amount of phubbing behaviors [29], and the effects of phubbing [4]. Therefore, gender differences in the moderated mediation model will also be explored.

### The present study

**Fig. 1:**
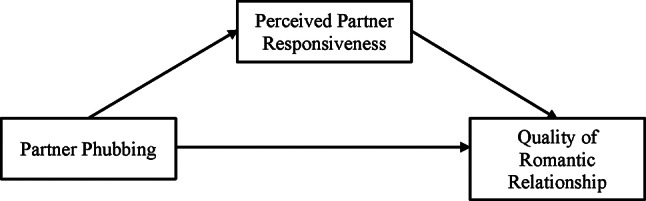
The proposed mediation model

**Fig. 2:**
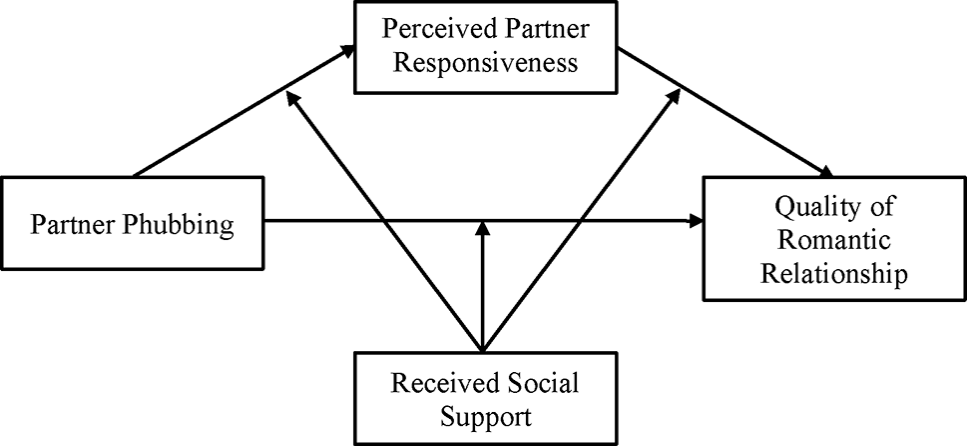
The proposed moderated mediation model

The current study attempts to examine a moderated mediation model of partner phubbing, PPR, RSS, and QR in emerging adults with a romantic partner in China. Chinese cultures value collectivism and belongingness to a group, which may make phubbing behavior and RSS more salient and influential in Chinese couples [30]. It is hypothesized that (1) partner phubbing would have an adverse association with QR. (2) PPR would mediate the association between partner phubbing and QR (Fig. 1). (3) RSS would moderate the mediation model (Fig. 2). Specifically, higher levels of RSS were expected to buffer the negative impact of partner phubbing on both PPR and QR, and to strengthen the positive association between PPR and QR. In addition, gender differences in the moderated mediation model will also be explored.

## Method

### Participants and procedures

Information was gathered from young adults using the Wen Juan Xing (www.wjx.cn) online survey platform in February 2021. The eligibility requirements comprised of (1) aged between 18 and 35 years old; (2) speaking Chinese; (3) using a smartphone on a daily basis; and (4) currently being in a regular romantic relationship. Previous research has reported differences in the levels of phubbing behaviors between married and non-married people [18]. Moreover, marriage is often associated with stronger relational investments, clearer long-term expectations, and greater societal and institutional support. As a result, married individuals generally report higher levels of relationship quality compared to their unmarried counterparts [31, 32]. Emerging adults in unmarried relationships may still be in the process of exploring their identities and future goals, which can introduce unique stressors and impact relationship quality differently than in more established marital relationships [5]. Given these distinctions, individuals who were married were excluded from our sample to ensure a more homogenous group reflective of the developmental characteristics of emerging adulthood. Participants were notified that participation was voluntary and anonymous, and that only the research team could access their data. Participants’ written informed consents were obtained. The study procedures were carried out in accordance with the Declaration of Helsinki. Ethics approval was obtained from the ethics committee of the corresponding authors’ institution. Participants would receive RMB 5 (about 0.77 USD) after completing the online questionnaire.

Of the 833 responses, 62 were excluded because they incorrectly answered the attention check questions (e.g., “Please select ‘Never’ for this question”). The remaining 772 participants (92.6%) were included in data analyses. Table 1 shows the background and psychosocial characteristics of the participants by sex. Most of the participants were female (65.0%), aged 19–24 years (88.3%), and had obtained an educational level of college or above (96.7%). Around 49.6% of them had dated for more than one year. More than half of the participants (58.8%) used smartphones at least 6 h per day. Females reported more hours per day of using smartphones than males (*χ*^2^ = 46.45, *p* < .001).

The mean (*SD*) of partner phubbing, RSS, PPR, and QR were 27.33 (5.82), 52.49 (10.50), 46.69 (7.56), and 33.41 (6.89), respectively. Males reported a higher level of QR than females (*t* = 3.28, *p* < .01).

**Table 1:** Descriptive statistics of all variables

	Total, *N* = 772	Male, *N* = 270	Female, *N* = 502	χ^2^/t-test
	*n* (%)/*Mean (SD)*	*n* (%)/*Mean (SD)*	*n* (%)/*Mean (SD)*	
**Background variables**				
Age (years)	21.54 (3.07)	21.75 (3.02)	21.43 (3.09)	1.36
≤ 18	54 (7.0)	16 (5.9)	38 (7.6)	60.14^***^
19 ∼ 20	287 (37.2)	87 (32.2)	200 (39.8)	
21 ∼ 22	233 (30.2)	88 (32.6)	145 (28.9)	
23 ∼ 24	108 (14.0)	50 (18.6)	58 (11.5)	
≥ 25	90(11.7)	29 (10.7)	61 (12.2)	
Hukou status				
Urban	530 (68.7)	189 (70.0)	343 (67.9)	0.54
Rural	242 (31.3)	81 (30.0)	161 (32.1)	
One-child family				
One-child	313 (40.5)	137 (50.7)	176 (35.1)	3.95
More than one child	459 (59.5)	133 (49.3)	326 (64.9)	
Education				
Middle school and below	2 (0.3)	-	2 (0.4)	4.48
High school	23 (3.0)	6 (2.2)	17 (3.4)	
College	648 (83.9)	121 (85.9)	416 (82.9)	
Postgraduate	99 (12.8)	32 (11.9)	67 (13.3)	
Smartphone using time/day				
Less than 2 h	8 (1.0)	7 (2.6)	1 (0.2)	46.45^***^
2 ∼ 4 h	76 (9.8)	34 (12.6)	42 (8.4)	
4 ∼ 6 h	234 (30.3)	94 (34.8)	140 (27.9)	
6 ∼ 8 h	256 (33.2)	77 (28.5)	179 (35.7)	
More than 8 h	198 (25.6)	58 (21.5)	140 (27.9)	
RD	4.00 (1.57)	3.96 (1.59)	4.02 (1.56)	− 0.46
Less than 1 month	58 (7.5)	24 (8.9)	34 (6.8)	2.70
1–3 months	122 (15.8)	40 (14.8)	82 (16.3)	
3–6 months	101 (13.1)	36 (13.3)	65 (12.9)	
6 months − 1 year	108 (14.0)	37 (13.7)	71 (14.1)	
1 year − 3 years	248 (32.1)	88 (32.6)	160 (31.9)	
More than 3 years	135 (17.5)	45 (16.7)	90 (17.9)	
**Psychosocial variables**				
Partner phubbing	27.33 (5.82)	26.90 (6.05)	27.57 (5.68)	-1.52
RSS	52.49 (10.50)	53.02 (10.80)	52.20 (10.33)	1.05
PPR	46.69 (7.56)	46.76 (8.05)	46.66 (7.29)	0.19
QR	33.41 (6.89)	34.51 (7.38)	32.81 (6.55)	3.28^**^

Note. *SD* = Standard Deviation, RD = Relationship Duration, RSS = Received Social Support, PPR = Perceived Partner Responsiveness, QR = Quality of Romantic Relationship. ^**^p < .01, ^***^p < .001

### Measures

#### Partner phubbing scale

The assessment of partner phubbing in a romantic relationship was conducted using the 9-item Partner Phubbing Scale developed by Roberts and David [10]. A five-point Likert scale was used, ranging from “Never” [1] to “All the Time” [5]. A higher total score reflects a greater frequency of being phubbed by their partner in a romantic relationship. A good reliability and validity were found in the Chinese version of the scale [33]. The Cronbach’s α was 0.76.

#### The significant others scale (SOS)

RSS from partners in romantic relationships was assessed by the 10-item Significant Others Scale (SOS), which was originally developed to assess received support from important others, including partners [34]. The SOS includes two categories of RSS: emotional support and practical support, with five items for each category rated on a 1–7 scale (1 = never to 7 = always). The scale in Chinese showed good reliability and validity [35]. The Cronbach’s α was 0.92.

#### Perceived partner responsiveness scale

The 12-item Perceived Partner Responsiveness Scale [36] was used to evaluate how much people believe they are being understood, respected, and cared for by their partners. A five-point Likert scale was used (1 = strongly disagree to 5 = strongly agree). The cumulative scores were computed by adding up responses to all the items, where a higher result indicates a greater perception of partner responsiveness. The Perceived Partner Responsiveness Scale was validated in the Chinese population [37]. The Cronbach’s α was 0.94.

#### The quality of relationship index

Romantic relationship quality was assessed by Quality of Relationship Index (QRI) [38], adapted from the Quality of Marriage Index [39]. The QRI consists of six items that assess the extent to which individuals are satisfied and happy with their relationship (e.g., “My relationship with my partner makes me happy”). The items were rated by a 7-point Likert scale (1 = strongly disagree to 7 = strongly agree). Higher total scores indicate better perceived romantic relationship quality. The Quality of Relationship Index in Chinese showed good reliability and validity [40]. The Cronbach’s α was 0.94.

#### Background factors

Background factors, including age, sex, education, Hukou status (a government system of household registration), one-child family, smartphone use time (hours per day), and relationship duration, were reported by the participants.

### Data analyses

Descriptive statistics, including mean, standard deviation (*SD*), and frequency, were presented. *Chi*-square tests or *t*-tests were used to compare the levels of variables between males and females. Pearson correlation coefficients among the variables were conducted. The variance inflation factor (VIF) for all the variables was calculated and the VIF values were below the common threshold of 5 suggesting that multicollinearity is not a significant concern for regression analysis [41]. The background factors of QR (including age, education, Hukou status, one-child family, smartphone use time (hours per day), and RD) were selected by simple linear regression analyses. The significant background factors were adjusted for in the subsequent mediation and moderation analyses. The mediation role of PPR and the moderation role of RSS were analyzed by Hayes’s PROCESS macro for SPSS [42]. Specifically, Model 4 in PROCESS was utilized to test the mediation hypothesis (Fig. 1). The size of the mediation effect (the proportion of mediation [*PM*]) was reported. Model 59 in PROCESS was employed to examine how RSS would moderate the mediation model (Fig. 2). The significance of the interaction term was evaluated and tested by using the change of *F-*values. The bootstrap confidence intervals (95% CIs without zero reflected statistical significance) were used to evaluate the significance of the effects in Model 4 and Model 59 by analyzing 5000 random samples [42]. Variables were standardized before the analysis. All analyses were stratified by gender. The level of statistical significance was 0.05. The analyses were conducted using SPSS version 26.0.

## Result

### Correlations among the Variables

Correlations among variables are presented in Table 2. Partner phubbing was negatively correlated to RSS (*r* = -.17, *p* < .01), PPR (*r* = -.27, *p* < .01) and QR (*r* = -.24, *p* < .01) in female participants. For both male and female participants, RSS was positively correlated to PPR (males: *r* = .74, *p* < .01; females: *r* = .63, *p* < .01) and QR (males: *r* = .75, *p* < .01; females: *r* = .70, *p* < .01). A significant correlation between PPR and QR was also found (males: *r* = .74, *p* < .01; females: *r* = .64, *p* < .01).

**Table 2:** Correlations among the variables

	1	2	3	4	5	6
1. Age	-	0.21^**^	0.06	0.01	0.01	− 0.03
2. RD	0.28^**^	-	0.07	0.22^**^	0.12^**^	0.19^**^
3. Partner phubbing	0.09	0.15^*^	-	− 0.17^**^	− 0.27^**^	− 0.24^**^
4. RSS	0.004	0.21^**^	− 0.05	-	0.63^**^	0.70^**^
5. PPR	0.004	0.16^**^	− 0.04	0.74^**^	-	0.64^**^
6. QR	0.25^**^	0.25^**^	− 0.07	0.75^**^	0.74^**^	-

Note. RD = Relationship Duration, RSS = Received Social Support, PPR = Perceived Partner Responsiveness, QR = Quality of Romantic Relationship. Males: below the diagonal; Females: above the diagonal. *p < .05; **p < .01

### Mediation effect of perceived partner responsiveness

For female participants, the total effect of partner phubbing on QR was negative and significant (*β*_total_ = − 0.24, 95%CI = [-0.33, − 0.16]). PPR significantly mediated the association between partner phubbing and QR (*β*_indirect_ = − 0.17, 95%CI = [-0.24, − 0.10], *PM* = 70.8%). The direct effect of partner phubbing on QR remained significant (*β*_direct_ = − 0.08, 95%CI = [-0.14, − 0.01]); Table 3).

For male participants, the total effect of partner phubbing on QR was non-significant. PPR was not a significant mediator in the association between partner phubbing and QR.

**Table 3:** The results of the mediation model by gender (*N*_male_=270, *N*_female_=502)

Predictors	QR		PPR		QR	
	Male	Female	Male	Female	Male	Female
	β [95% CI]	β [95% CI]	β [95% CI]	β [95% CI]	β [95% CI]	β [95% CI]
Partner phubbing	− 0.11 [-0.23, 0.01]	− 0.24 [-0.33, − 0.16]	− 0.07 [-0.19, 0.06]	− 0.28 [-0.36, − 0.20]	− 0.07 [-0.15, 0.02]	− 0.08 [-0.15, − 0.01]
PPR			-	-	0.71 [0.64, 0.80]	0.60 [0.53, 0.66]
*R* ^*2*^	0.07	0.11	0.03	0.09	0.58	0.44
*F*	7.13^***^	19.47^***^	2.46	16.70^***^	89.77^***^	96.27^***^

Note. RD = Relationship Duration, PPR = Perceived Partner Responsiveness, QR = Quality of Romantic Relationship. *β* = Standardized linear regression coefficient. 95% CI = 95% Confidence Interval. Age and RD were the control variables. ***p < .001

### Moderated mediation effect analysis

**Fig. 3:**
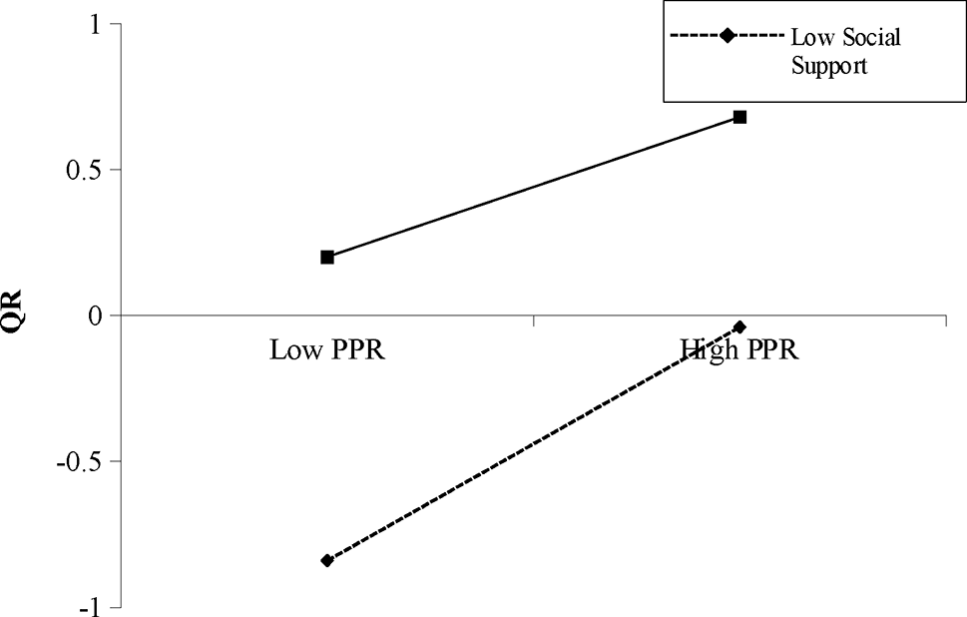
Interaction between PPR and RSS on QR for female participants

For females, controlling for background factors (i.e., age and RD), the interaction term of PPR and RSS was significantly associated with QR (*β*=-0.08, *p* < .001, 95%CI [-0.13, − 0.03]) (Table 4). Simple slope test (Fig. 3) showed that the positive association between PPR and QR was stronger when RSS was low (mean-SD) (*B*_simple_ = 0.40, *p* < .001) than that when RSS was high (mean + *SD*) (*B*_simple_ = 0.25, *p* < .001). The moderated mediation effect was not significant.

**Fig. 4:**
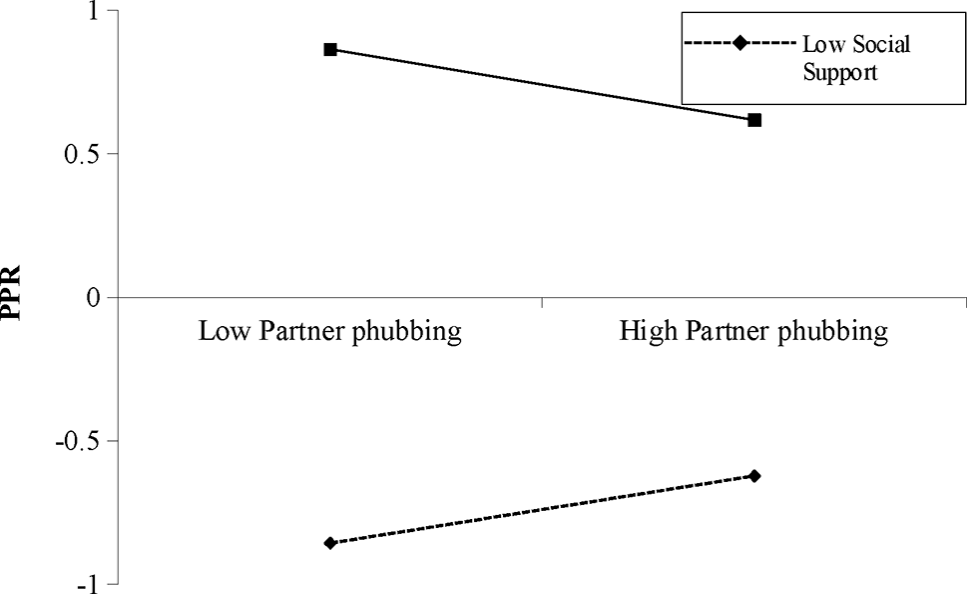
Interaction between partner phubbing and RSS on PPR for male participants

**Fig. 5:**
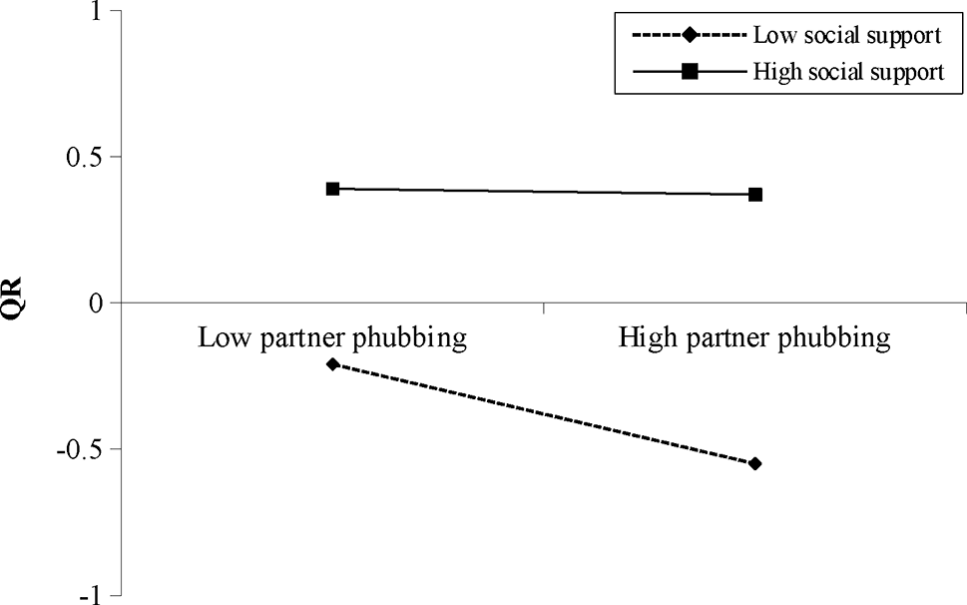
Interaction between partner phubbing and RSS on QR for male participants

**Fig. 6:**
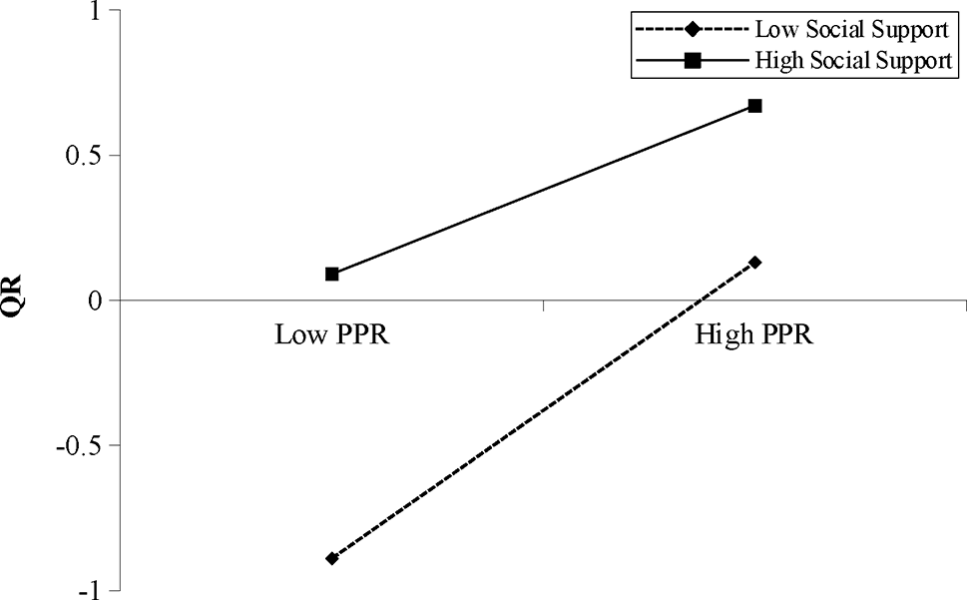
Interaction between PPR and RSS on QR for male participants

For male participants, although partner phubbing and PPR were not significantly associated, such association was negatively moderated by RSS (Table 4). Simple slope test (Fig. 4) showed that partner phubbing was negatively associated with PPR when the RSS was high (*B*_simple_ = − 0.13, *p* < .05). But when the RSS was low, partner phubbing became positively associated with PPR (*B*_simple_ = 0.11, *p* < .05). The negative association between partner phubbing and QR was positively moderated by RSS (*β* = 0.08, *p* < .05, 95%CI [0.01, 0.15]). Simple slope test (Fig. 5) showed that partner phubbing was negatively associated with QR when the RSS was low (*B*_simple_ = − 0.17, *p* < .01). Such the association became non-significant when RSS was high. In addition, the interaction term of PPR and RSS was significantly associated with QR (*β*=-0.11, *p* < .001, 95%CI [-0.16, − 0.07]). Simple slope test (Fig. 6) showed that the positive association between PPR and QR was stronger when RSS was low (*B*_simple_ = 0.51, *p* < .001) than when RSS was high (*B*_simple_ = 0.28, *p* < .001). The moderated mediation effect was not significant.

**Table 4:** The results of the moderated mediation model by gender (*N*_male_=270, *N*_female_=502)

Predictors	PPR		QR	
	Male	Female	Male	Female
	β [95% CI]	β [95% CI]	β [95% CI]	β [95% CI]
Age	− 0.003 [-0.03, 0.03]	− 0.01 [-0.02, 0.02]	0.01 [-0.02,0.03]	− 0.01 [-0.03,0.01]
RD	− 0.001 [-0.06, 0.06]	− 0.01 [-0.05, 0.03]	0.07 [0.02, 0.12]	0.04 [0.01, 0.08]
Partner phubbing [X]	− 0.003 [-0.09, 0.08]	**− 0.17 [-0.24**,** − 0.11]**	**− 0.09 [-0.17**,** − 0.02]**	**− 0.07 [-0.13**,** − 0.02]**
RSS [W]	**0.74 [0.66**,** 0.83]**	**0.59 [0.52**,** 0.65]**	**0.38 [0.26**,** 0.49]**	**0.44 [0.36**,** 0.51]**
X × W	**− 0.12 [-0.19**,**-0.05]**	− 0.01 [-0.07, 0.06]	**0.08 [0.01**,** 0.15]**	− 0.05 [-0.11, 0.01]
PPR [M]			**0.40 [0.30**,** 0.51]**	**0.32 [0.25**,** 0.40]**
M × W			**− 0.11 [-0.16**,** − 0.07]**	**− 0.08 [-0.13**,** − 0.03]**
*R* ^*2*^	0.58	0.42	0.67	0.58
*F*	72.15^***^	71.94^***^	77.30^***^	98.10^***^
*ΔR*^2^ [X × W]	0.020	< 0.001	0.007	0.002
*F* [X × W]	12.28^***^	0.08	5.33^*^	2.32
*ΔR*^2^ [M × W]			0.028	0.009
*F* [M × W]			22.13^***^	10.85^**^

Note. RD = Relationship Duration, RSS = Received Social Support, PPR = Perceived Partner Responsiveness, QR = Quality of Romantic Relationship. *β* = Standardized linear regression coefficient. 95% CI = 95% Confidence Interval. Bold indicates statistically significant results. Age and RD were the control variables. *p < .05, **p < .01, ***p < .001

## Discussion

This is the first study to test both PPR and RSS as potential mechanisms in the association between partner phubbing and QR. Our hypotheses were partially supported, while some intriguing findings were reported.

### Mediation effect of PPR

PPR was a significant mediator between partner phubbing and QR in females but not in males. This aligns with earlier research indicating that PPR plays a critical role in romantic satisfaction [10, 12, 13] and extends it by suggesting this pathway may operate differently across genders. One explanation may lie in gender role theory [27], which suggests women are socialized to value emotional closeness and interpersonal harmony more than men. This makes them more attentive to their partner’s responsiveness—and therefore more affected by disruptions such as phubbing. Cultural context may further amplify this gender difference. In collectivist societies like China, women are often socialized to invest heavily in maintaining relationship harmony, driven by Confucian norms emphasizing female loyalty and submission, resulting in a higher cost of divorce/breaking up for women than for men [43]. This may lead women to be more vigilant to perceived relational threats like phubbing, which undermines responsiveness. By contrast, the non-significant mediation among males suggests that men may interpret or respond to phubbing differently. Prior studies [2] found that men reported fewer relationship conflicts in response to partner phone use, suggesting they may be less sensitive to this type of digital interruption.

Although the mediation effects were modest, the results emphasize the importance of maintaining responsiveness in digital-era relationships. It is important for couples to recognize the potential negative effects of phubbing behavior [44], and enhance the quality of face-to-face interactions in order to improve couples’ QR [45]. The gender difference indicates that women might be more vulnerable to phubbing than men, though, for both genders, PPR was a strong indicator of their QR. Therefore, increasing one’s responsiveness through intervention and training could be an effective way to protect partners’ QR. Some skill-training interventions for couples may be helpful. For instance, consistently practicing high-quality listening can enhance couples’ PPR and QR [46]. Such skills require couples to show that they understand and value their partners’ thoughts and needs when listening, which can be conveyed through facial expressions, follow-up questions, gestures, and eye contact during interactions.

Additionally, it should also be noted that the influence of partner phubbing on PPR and QR was relatively small. Çizmeci [18] provided a possible explanation by emphasizing the role of normalization; that is, phubbing could be a normalized behavior among young people, thus leading to a relatively small effect on relationships and interpersonal interactions. In other words, the phubbing behavior may be more acceptable to young couples, which could result in a kind of social exchange behavior, i.e., phubbing toward each other [13]. Future studies might compare these effects between young couples and older couples.

### Moderation effect of RSS

RSS was positively associated with PPR and QR, yet paradoxically weakened the association between PPR and QR across genders. This may be explained by the Vulnerability-Stress-Adaptation model [47], which posits that the effect of adaptive processes (e.g., responsiveness) on relationship outcomes depends on the broader social and contextual stressors. When external RSS is high, the role of responsiveness may become less central, while low support may heighten sensitivity to partner behaviors [48].

Intriguingly, for males, partner phubbing was negatively associated with PPR when RSS was high, and the association became positive when RSS was low. One hypothesis is that among low-support male receivers, the appraisal of phubbing behavior was different from high-support receivers. Conflict tends to be more prevalent among partners experiencing low RSS, which may also lead to lower expectations of their partners. According to Panova and Lleras [49], phubbing can also be evaluated as a response to passive-aggressively express one’s dissatisfaction, or withdrawal from the scenario. Despite its unsatisfactory nature, males with low expectations might interpret phubbing as a response to their demand, or as a concession to avoid escalated tensions. Therefore, they may perceive their partner’s action as an indication of valuing the relationship and acknowledgment of their needs. However, the precise role of conflict in shaping the effect of phubbing warrants further investigation. Additionally, it is important to acknowledge that this unexpected result may also reflect a suppression effect or statistical artifact due to overlapping variance or sample-specific characteristics. Therefore, future research endeavors should seek to validate these findings through more extensive and inclusive sampling methodologies.

For females, RSS did not compensate for the adverse effect of partner phubbing. This may be partly related to the different types of RSS that males and females provide to their partners. Jackson [50] argued that one’s RSS would be effective when the type of support matches the recipient’s needs. However, another key factor is whether RSS is seen as responsive or unsolicited by the recipient. According to MacGeorge, Feng [51], social gender roles encourage females to provide more emotional support to their partners, while males tend to provide more instrumental support. The latter may be less effective or even stress-inducing in addressing emotional exclusion experienced by females during phubbing. Furthermore, even when males’ support matches their partner’s needs, if it is perceived as unsolicited, it may still fail to mitigate the psychological impact of being phubbed. In line with this reasoning, in order to buffer the stress of social exclusion caused by partner phubbing, more training in providing emotional support would be needed.

The non-significant moderated mediation effects observed in our sample may suggest that the moderating role of RSS is limited to specific paths within the model and is not strong enough to yield a significant conditional indirect effect overall. Future research should explore alternative moderators, such as attachment style or rejection sensitivity, that may influence the strength of the indirect effect of PPR on QR.

### Theoretical implications

This study advances the theoretical understanding of romantic relationship functioning in emerging adulthood by revealing gender-specific pathways through which partner phubbing impacts QR. These different patterns cross gender align with developmental theories suggesting that emerging adults are still refining their emotional regulation and intimacy skills [5, 52]. The findings also support expanding relational models to account for technological intrusions in daily interactions, offering a more nuanced framework for digital-age relationship maintenance. By identifying both mediating and moderating processes, this study provides a more nuanced framework for understanding how partner phubbing undermines relational well-being during a critical life stage marked by identity exploration and interpersonal growth.

### Limitations

This study had several limitations. First, given that it was a cross-sectional design, one should be cautious about the conclusions regarding causal relationships among the mediation models. It is possible that low QR leads to reduced interest in couples’ interactions, which results in more phubbing behavior and less PPR [9]. Second, since self-reported questionnaires were used, self-report bias may exist. Third, the reciprocal nature of phubbing was not considered. Reciprocity means that when one of the partners starts to phub, the phubbee may increase the same behavior as a response [13]. Ahlstrom, Lundberg [53] found that people were less likely to report dissatisfaction when both partners played video games than when only one of them played. Fourth, since this was convenient sampling, sample bias may exist (e.g., a higher proportion of university-education participants and online users). Future work should validate the findings in a more diverse and representative sample to enhance the generalizability of the results. Lastly, although the moderation effects are statistically detectable, the magnitude of the effects is relatively modest, and the practical impact may be minor. Future research should explore alternative moderators to uncover more practically meaningful effects.

### Future directions

Future research should first consider longitudinal or experimental designs to clarify the causal pathways among partner phubbing, PPR, and QR. Second, examining actor-partner effects using dyadic data would deepen the understanding of mutual influences within couples. Third, since PPR only mediated the relationship between partner phubbing and QR among females, future studies should explore alternative mediators for both genders. Prior research has identified other relational processes, such as perceived exclusion, conflict, and jealousy, as potential mediators [12]. Moreover, experimental studies show that even imagined phubbing (e.g., in animations) can reduce perceived communication quality and relationship satisfaction [54]. These findings support the need to broaden the current model and investigate whether PPR mediates phubbing’s impact across different types of social interactions and relationships beyond romantic contexts. Fourth, although this study examined the moderation role of RSS, it is important to note that RSS is not universally associated with positive outcomes. Previous research indicates that the effects of RSS may vary depending on support type, context, and individual perception, and can sometimes be experienced as intrusive or undermining [55, 56]. Future studies should consider these nuances by examining moderators such as the perceived responsiveness of the support or distinguishing between emotional and instrumental support components.

## Conclusion

This study sheds light on the potential interpersonal implications of partner phubbing among young couples and examines the possible pathways through which partner phubbing can negatively impact the QR. PPR was found to be a significant mediator for female participants. Among males, RSS could buffer the negative effect of partner phubbing on QR. The direction of the effect of RSS on the relationship between partner phubbing and PPR among males was inconsistent with our original hypothesis and should be further explored. It would be beneficial for research to utilize a longitudinal approach and examine additional factors that may moderate or mediate the findings. Interventions to enhance awareness and reduce the negative effects of phubbing on couples should be promoted.

## Data Availability

The data that support the findings of this study are openly available in OSF at https://osf.io/6bfx4/?view_only=ea92475a788f4b609e424136aefe52c1.
